# Paediatric dental trauma: insights from epidemiological studies and management recommendations

**DOI:** 10.1186/s12903-024-05222-5

**Published:** 2025-01-02

**Authors:** Alessandra Laforgia, Angelo Michele Inchingolo, Francesco Inchingolo, Roberta Sardano, Irma Trilli, Angela Di Noia, Laura Ferrante, Andrea Palermo, Alessio Danilo Inchingolo, Gianna Dipalma

**Affiliations:** 1 Department of Interdisciplinary Medicine, University of Bari “Aldo Moro”, Bari, 70124 Italy; 2 Department of Experimental Medicine, University of Salento, Lecce, 73100 Italy

**Keywords:** Dental trauma, Children, Dental injuries, Dental fractures, Emergency treatment

## Abstract

Dental trauma is common in all age groups, although, epidemiologically, it is more common in children with studies that indicate that 15% of preschoolers and 20–25% of school-age children experience it. These injuries, which frequently call for immediate attention, can affect the hard tissues and supporting components of the teeth, and, because dental damage in deciduous teeth occurs frequently and affects speech, nutrition, and oral development, it is particularly worrying. After searching three databases, Scopus, Web of Science (WoS), and PubMed, and removing duplicates, 3,630 articles were screened, and 12 publications were included in the qualitative analysis. Due to their busy lifestyles, children are particularly susceptible to oral trauma and in certain areas and lower socioeconomic groups, the incidence is higher. From little fractures in the enamel to serious dislocations and avulsions, injuries vary and must be treated promptly in order to avoid consequences and to prevent long-term issues. Furthermore, a conservative treatment strategy is recommended to preserve tooth vitality and prevent extractions. Reducing the occurrence of dental injuries requires the implementation of preventive measures including mouthguard use and educational campaigns. In summary, this review emphasizes the importance of early diagnosis, immediate management, and long-term care, by synthesizing existing knowledge on the prevalence, types, management, complications, and prevention of dental trauma in deciduous teeth. Finally, it’s important to underscore the need for continued research to refine treatment approaches.

## Introduction

Dental trauma is a common event across all ages, with a particularly high prevalence in childhood [1–4]. Recent studies indicate that dental injuries are on the rise, driven by active lifestyles and increased participation in sports and outdoor activities among children [5]. A European study found that 15% of preschoolers and 20–25% of school-age children have experienced dental trauma [6]. These injuries can affect the hard tissues of the tooth (enamel, dentin, cementum) with or without pulp involvement, or the supporting tissues (bone, ligament, gingiva) [7–10]. Dental trauma is a distressing experience for both children and adults, often requiring immediate intervention to increase the chances of successful treatment, especially in the childhood [11–15]. The urgency of addressing dental trauma in pediatric dentistry is underscored by its potential to cause long-term complications and affect the development of permanent dentition [16–19]. Immediate and appropriate management can mitigate risks such as pulpal necrosis, infection, and damage to the developing permanent teeth. It’s crucial to reassure the child, as parental anxiety can sometimes exacerbate the child’s distress more than the trauma itself [20]. Injuries to deciduous teeth are of particular concern due to their crucial role in guiding the eruption of permanent teeth, maintaining space, and supporting proper speech and nutrition. Consequently, trauma to these teeth can have lasting impacts on a child’s dental and overall health [21–23]. The prevalence of dental trauma in children varies widely based on geographic location, socioeconomic status, and demographics. Recent data suggest that young children, due to their active lifestyles and developing motor skills, are especially vulnerable, with those from lower socioeconomic backgrounds and certain regions being at higher risk [24–28]. The types of dental trauma suffered by children can vary widely, ranging from minor enamel fractures to more serious injuries such as dislocation, avulsion, and crown fractures [1, 29–31]. Dislocation injuries, in which the tooth is displaced from its normal position, are particularly common in primary teeth because of their relatively less rigid support structures than in permanent teeth [7, 32, 33].

Immediate and appropriate management, with prompt intervention and follow-up care, of dental trauma is critical to improve outcomes and minimize complications [34–41]. The ‘conservative approach, whenever possible, to preserve the vitality of the injured tooth and avoid unnecessary extractions is always preferred [42–47].

Complications from dental trauma in primary teeth can be significant, with potential impacts on both the injured primary tooth and the developing permanent successor [48–51]. Treatments, using various methods, of direct pulp capping in traumatized primary teeth have been successful [52–56].

**Fig. 1:**
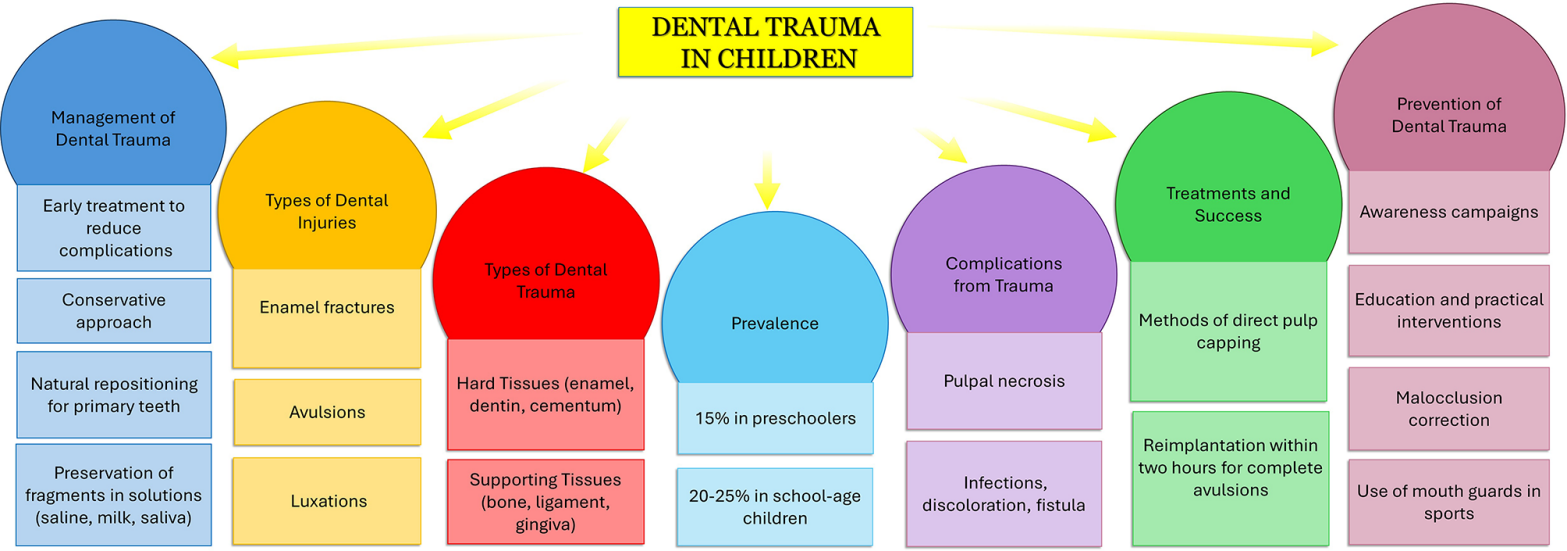
Concept map of dental trauma in children

If the deciduous tooth has been displaced from its original position, natural repositioning is expected [57–60]. If the trauma involves a permanent tooth and it has broken off, it is important to look for the fragment and preserve it in saline, milk or saliva [61–63]. Reattaching the fragment is often the most effective therapy [21, 64]. If the tooth has lost its vitality, root canal therapy will be necessary [65–68]. If the tooth is completely out of its socket (avulsion), it should be stored in an aqueous solution (saline, milk, or saliva) without cleaning it so as not to damage the periodontal fibers, and the dental clinic should be contacted immediately [69–74]. In the case of complete avulsions, reimplantation of the tooth within two hours may have a good chance of success [75, 76]. The traumatized tooth should be monitored for a long period of time to detect and limit any late damage, such as periodontal or pulpal damage [77–82]. Prevention of dental trauma is a key aspect of pediatric dental care, involving both education and practical interventions [83–85]. Targeted preventive strategies in vulnerable populations could reduce the overall prevalence. The use of mouth guards in preventing dental injuries among young athletes demonstrates the importance of protective clothing in high-risk activities [86–90]. Thus, there is a need for ongoing education and social awareness campaigns to reduce the incidence of dental trauma [91–97]. Despite the wealth of research on dental trauma in primary teeth, there remains a need for a comprehensive and up-to-date synthesis of the existing literature [11, 98–100]. This systematic review aims to consolidate current knowledge on the prevalence, types, management, complications, and prevention of dental trauma in deciduous teeth [9, 101–103], seeking to identify gaps in current understanding and providing recommendations for better prevention and management strategies with a look to a future research [58, 104–106] (Fig. 1).

## Materials and methods

### Protocol and registration

The Preferred Reporting Items for Systematic Reviews and Meta-Analysis (PRISMA) were followed in the conduct of this systematic review, which was then submitted to PROSPERO under the ID of CDR 566,811.

### Search processing

Using the keywords “dental trauma” and “children,” we searched databases as Scopus, Web of Science (WoS), and PubMed to find papers relevant to this topic. The search was limited to the last 15 years (May 2010–May 2024).

### Eligibility criteria

The reviewers, in a double-blind manner, included papers that satisfied the following criteria for inclusion: [1] articles related to trauma in children population and [2] clinical studies or case series.

Exclusion criteria were represented by reviews (systematic and/or narrative) with/without meta-analyses, studies regarding adult populations, animal models, and in vitro studies.

### Data processing

After the exclusion of any publications that did not fit the themes examined, the complete texts of the publications that had been included earlier were read as part of the screening process, which involved reviewing the article titles and abstracts selected in the previous identification step. A third reviewer (FI) was consulted in cases of dispute after the reviewers had discussed the chosen articles.

### Quality assessment

The quality of the included papers was assessed by two reviewers, R.S. and I.T., using ROBINS, a tool developed to assess the risk of bias in the results of non-randomized studies that compare the health effects of two or more interventions. Seven points were evaluated, and each was assigned a degree of bias. A third reviewer (F.I.) was consulted in the event of a disagreement until an agreement was reached.

## Results

**Fig. 2:**
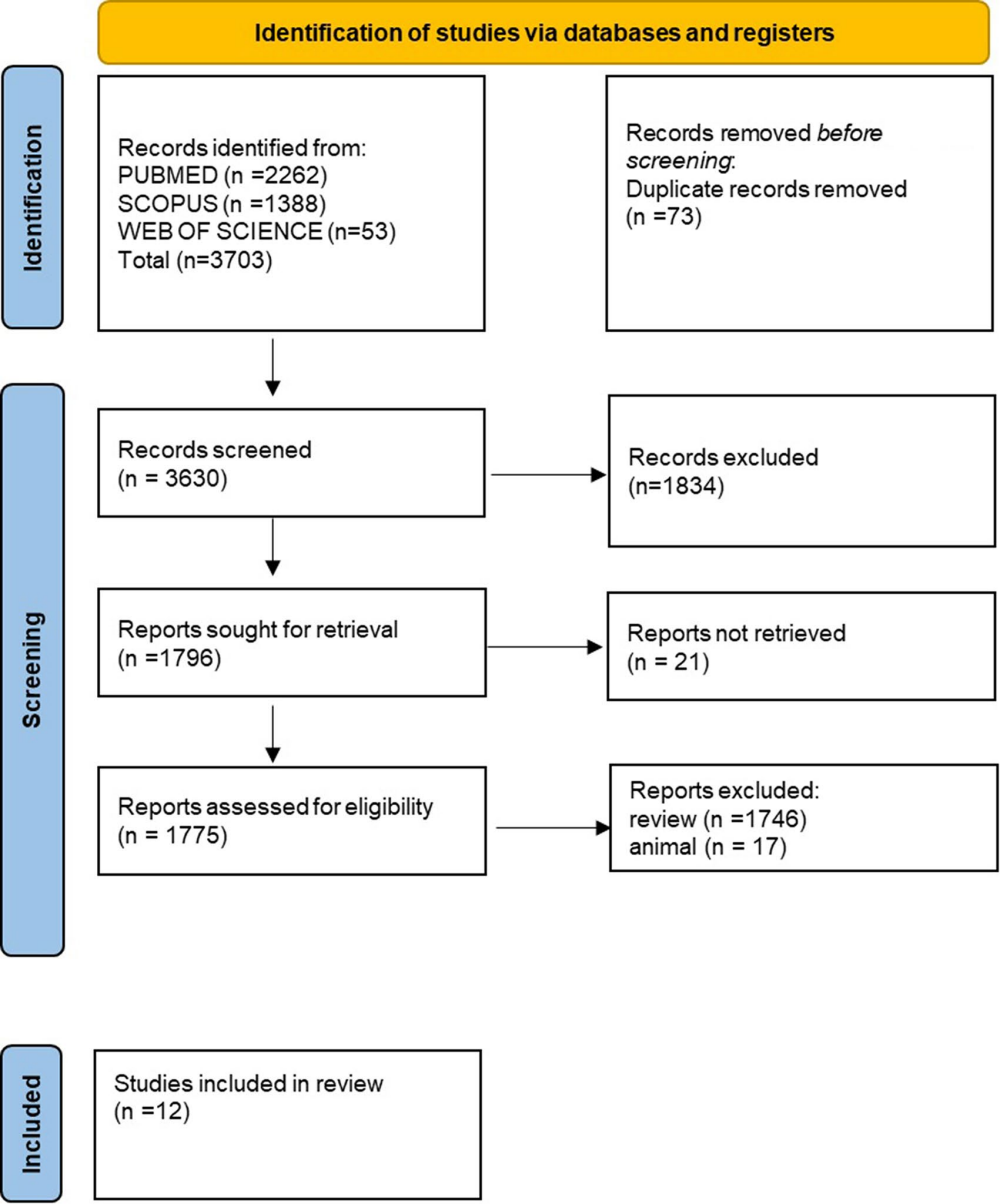
PRISMA flow diagram

3703 publications were found after searching four databases: Pubmed (2262), Web of Science [53], Scopus (1388). Following the elimination of duplicate entries [73], 3630 records underwent title and abstract screening, which resulted in the rejection of 1834 articles. After a full-text examination, 1784 were not included for not meeting the inclusion requirements. Ultimately, a total of 12 publications were deemed eligible for qualitative analysis (Table 1). Figure 2 provides an overview of the selecting procedure.

**Table 1:** Qualitative evaluation of the included studies

Authors (Year)	Type of the study	Aim of the study	Materials	Results
Kun Xuan et al., 2018 [107]	Randomized Controlled Clinical Trial.	The aim of the study is to evaluate the efficacy of implantation of autologous tooth stem cells from deciduous teeth (hDPSCs) in regenerating dental pulp and promoting root development in patients with pulp necrosis following traumatic dental injuries.	The study enrolled 40 patients with pulp necrosis post-traumatic dental injuries in a randomized, controlled trial. Thirty patients received hDPSC implantation, while ten received traditional apexification; four hDPSC patients were excluded due to follow-up loss or retrauma. Follow-up assessed hDPSC implantation safety and efficacy over 24 months.	The study demonstrates that implantation of hDPSCs effectively regenerates dental pulp, promotes root development, and shows potential as a treatment option for traumatic dental injuries causing pulp necrosis.
de Amorim et al., 2018 [108]	Retrospective Study	To identify the most common types of trauma to primary teeth	Data from 815 anterior primary teeth with dental injuries were collected from the records of 483 children aged 0–9 years at the time of trauma.	Root dilacerations were more frequent in children older than 3 years, while crown dilacerations were more common in children up to 3 years old. The primary treatments were tooth extraction and orthodontic treatment.
E. Bardellini et al., 2017 [109]	Retrospective study	To evaluate the prevalence of dental anomalies in permanent teeth due to trauma to primary teeth.	Analysis of 241 records of children (118 males, 123 females, mean age 3.62) with primary teeth trauma. Clinical and radiographic evaluations of the permanent successor teeth were conducted.	Intrusive and extrusive luxation were the most associated with clinical disturbances in successor teeth.
G. Martioli et al., 2019 [110]	Longitudinal Study	To evaluate the clinical and radiographic sequelae of dental trauma to primary teeth in children and its effects on permanent successors.	150 children treated for dental trauma to primary teeth.	The majority of injuries were uncomplicated crown fractures and periodontal tissue lesions, particularly lateral luxation.
Y.T. Yang et al., 2020 [111]	Randomized Prospective Controlled Trial	To evaluate the clinical and radiographic outcomes of using Innovative BioCeramix (iRoot BP Plus, Vancouver, BC), for partial pulpotomy in immature permanent teeth with complicated crown or crown-root fractures.	110 immature permanent teeth with complicated fractures were randomly assigned to two groups (*n* = 55): iRoot BP Plus (experimental) and calcium hydroxide (control).	The calcific bridge was significantly thinner in the iRoot BP Plus group compared to the calcium hydroxide group.
Q. Rao et al., 2020 [112]	Retrospective Study	To compare iRoot BP Plus to calcium hydroxide for pulpotomy in permanent incisors with complicated crown fractures.	Reparative dentin bridges and pulp canal obliteration were analyzed.	Reparative dentin bridges formed in 92.4% of the iRoot BP Plus group and 90% of the calcium hydroxide group. Pulp canal obliteration occurred in 2% of teeth in both groups.
A. J. H. Huh et al., 2022 [90]	Randomized Controlled Trial	To evaluate if a clinical decision support tool (CDST) enhances dental trauma knowledge in medical students and pediatric dentists, and to compare the effectiveness of print and mobile app CDSTs.	Medical students (*n* = 100) and pediatric dentists (*n* = 49) took a pretest to measure baseline dental trauma knowledge.	Participants using the mobile app CDST scored highest on the posttest but took the longest time to complete it.
Goswami et al., 2021 [113]	Retrospective study	To evaluate the prevalence of dental trauma in children in New Delhi	6,765 children between 1 and 14 years of age were evaluated for age, gender, type of trauma, and involvement of soft tissue	A significantly high prevalence of dental trauma is found in the anterior teeth
Diaz et al., 2010 [114]	Cross-sectional study	To identify the aetiology, types of traumatic dental injuries in primary and permanent dentitions, sex and age distributions, accident location in Temuco, Chile	359 patients, aged 1–15 years, with 145 primary teeth and 525 permanent teeth affected by dental trauma	The most common injuries were subluxation and avulsion in primary teeth, and crown fractures in permanent teeth
Patidar et al., 2021 [115]	Retrospective study	To analyze dental traumatic injuries and their management in children	466 children under 16 years	Ellis class IV fracture was noted as the most frequent type of dental injury and fall was a major etiological factor
Basha et al., 2021 [116]	Analytical cross-sectional study	To assess the association between traumatic dental injuries (TDIs) and obesity in children with special health care needs	350 (131 boys and 219 girls) special needs children with a median age of 12.0 years	There was a significant association between TDI prevalence and increased overjet, inadequate lip coverage, and cerebral palsy
Aldrigui et al., 2011 [117]	Cross-sectional study	To assess the impact of traumatic dental injuries and anterior malocclusion traits on the Oral Health-Related Quality of Life (OHRQoL) of children in Brazil	260 preschool children between 2 and 5 years-old	The presence of oral diseases and disorders can produce an impact on the quality of life of preschool children and their parents

### Quality assessment and risk of bias of included articles

**Fig. 3:**
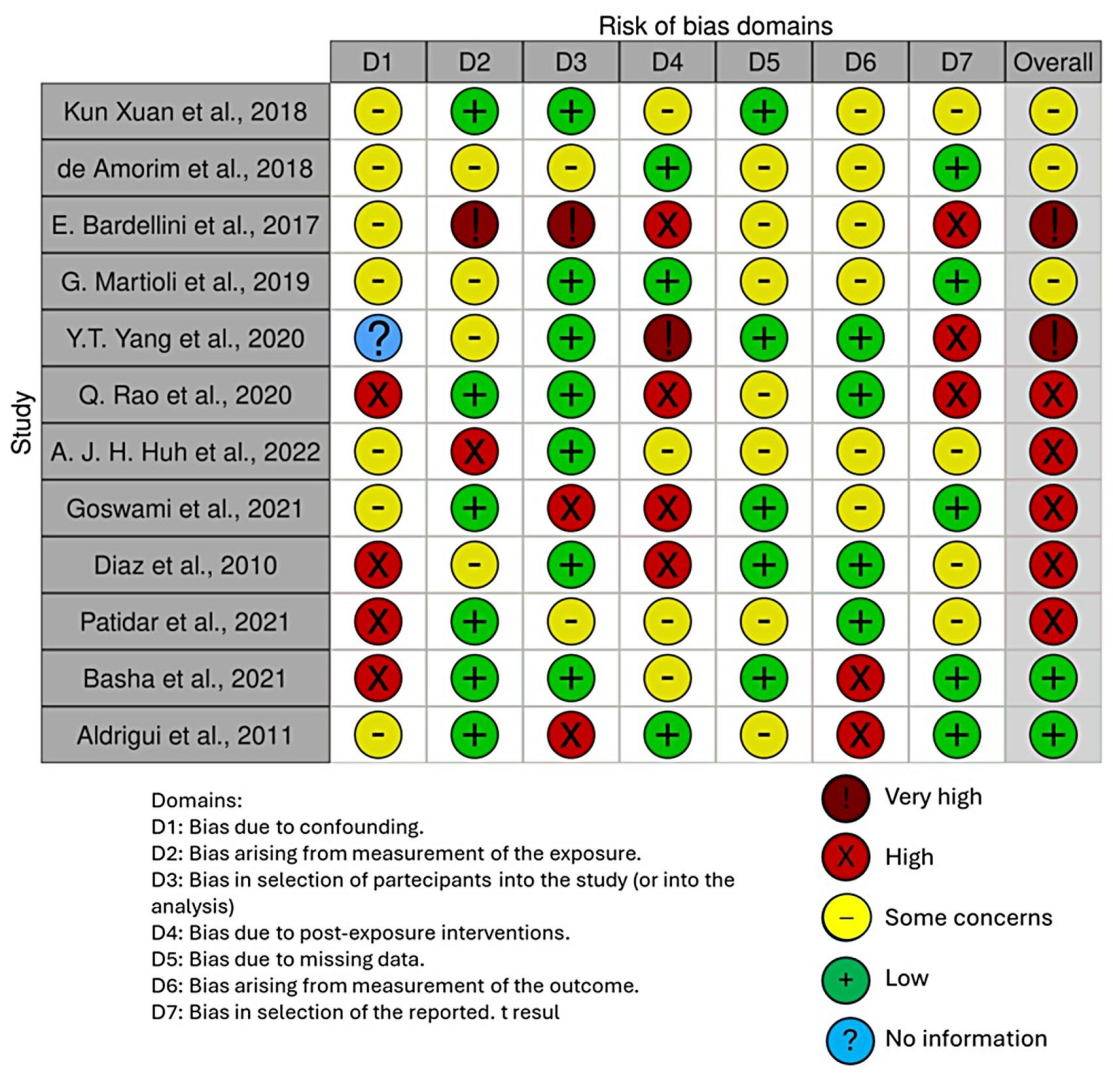
Bias assessment

The risk of bias in the included studies is reported in Fig. 3. Regarding the bias due to confounding most studies have a high risk. The bias arising from measurement is a parameter with low risk of bias. Many studies have low risk of bias due to bias in selection of participants. Bias due to post exposure cannot be calculated due to high heterogeneity. The bias due to missing data is low in many studies. Bias arising from measurement of the outcome is low. Bias in the selection of the reported results is high in most studies. The final results show that 4 studies have low risk of bias, 3 have a very high risk of bias and 5 have high risk of bias.

## Discussion

Dental trauma, particularly in immature teeth, presents significant clinical challenges due to the incomplete development of roots and the high risk of pulp necrosis. This condition can severely weaken teeth, complicating traditional endodontic treatments [107]. Traditional methods often fail to provide optimal outcomes, leading to issues such as thin canal walls and further dental problems. Consequently, revitalization techniques have gained attention for their potential to regenerate dental pulp and dentin, promoting root development and long-term tooth viability.

### 4.1 Epidemiology of Dental Trauma

Epidemiological studies provide valuable data on the prevalence and causes of dental trauma in children.

Epidemiological studies by Goswami et al., Diaz et al., Patidar et al., Basha et al., and Aldrigui et al. provided valuable data and management recommendations for dental trauma in children. These studies highlighted the prevalence of dental injuries, common causes, and the importance of timely treatment and education. For instance, Diaz et al. and Patidar et al. both identified falls and sports injuries as primary causes of dental trauma, emphasizing the need for immediate care and proper management to prevent long-term complications [90, 108]. Goswami et al. conducted a retrospective study evaluating dental trauma in 6,765 children aged 1–14, finding a prevalence of 1.25% with males being more affected. The most common injury was Ellis type IV fractures, predominantly affecting maxillary central incisors, with nearly half of the cases involving soft tissue injuries. This study emphasizes the need for preventive dental care and educational programs to manage and prevent dental injuries in children [109]. Diaz et al.‘s cross-sectional study, involved 359 pediatric patients treated for dental trauma, revealing that 37.9% of dental emergencies in children were due to trauma. Boys aged 7–12 were the most affected, with falls, object strikes, and bike accidents being the leading causes. The most common injuries were subluxation and avulsion in primary teeth, and crown fractures in permanent teeth. Maxillary incisors were the most frequently affected teeth in both dentitions. A significant number of patients received delayed emergency care, highlighting the need for better education on immediate replantation and proper handling of avulsed teeth [110]. Patidar et al.‘s retrospective analysis of data from 466 children under 16 years who sustained traumatic dental injuries emphasized the higher incidence of dental trauma in the permanent dentition group, with males predominantly affected. Soft tissue injuries were common, particularly in the primary dentition group [111–115]. Management primarily involved root canal treatment for permanent teeth, while primary teeth injuries were mainly managed by observation and wound care [116, 117]. Accidental falls were the most frequent cause of dental injuries, highlighting the need for timely and appropriate management to prevent complications and educating parents and teachers on prevention and immediate response to dental trauma [118]. Basha et al.‘s study on the prevalence of traumatic dental injuries among special needs children found that 23.1% of children experienced traumatic dental injuries (TDI), with falls and striking against objects being the main causes. Obesity and inadequate lip coverage were associated with higher TDI prevalence in bivariate analysis, but not in multivariate analysis [119, 120]. Cerebral palsy was significantly linked to higher TDI risk [121]. Most injuries occurred at school, indicating a need for increased oral health education for teachers and parents. Only 23.1% sought dental treatment, highlighting the need for better awareness and trained professionals [122].

### Diagnosis and management

Early and accurate diagnosis is crucial in mitigating long-term dental anomalies. Further exploring the impact of early dental trauma on the development of permanent teeth, studies by C.S. de Amorim (2018) and E. Bardellini (2017) emphasized the long-term consequences of such injuries. Amorim’s study found a notable incidence of crown and root dilaceration in permanent incisors following trauma to their primary predecessors [123]. Intrusive luxations and avulsions were the most frequent traumas associated with these anomalies. Bardellini’s retrospective study similarly noted that intrusive luxations were a significant risk factor for enamel hypoplasia and eruption disturbances in permanent teeth. Both studies underscore the importance of early and accurate diagnosis and intervention to mitigate long-term dental anomalies [124]. G. Martioli’s (2019) longitudinal study provided insights into the long-term effects of dental trauma on both deciduous and permanent teeth. The study involved 150 children and highlighted the correlation between the age at trauma and the severity of subsequent dental issues. It was found that early trauma often led to enamel hypoplasia and other complications in permanent teeth. This underscores the need for regular follow-ups and prompt treatment to prevent or minimize the sequelae of dental injuries [125].

### Treatment innovations

In terms of treatment, comparative studies by Y.T. Yang (2020) and Q. Rao (2020) focused on the efficacy of different pulpotomy materials in managing traumatized immature permanent teeth. Yang’s randomized controlled trial compared Innovative BioCeramix (iRoot BP Plus, Vancouver, BC), with traditional calcium hydroxide (CH), revealing a higher pulp survival rate with iRoot BP Plus, although the difference was not statistically significant [126]. Rao’s retrospective study further supported iRoot BP Plus as a promising alternative due to its higher success rate and easier handling compared to CH. These findings advocate for the adoption of newer materials that offer better sealing properties and ease of use [127]. Kun Xuan et al. (2018) conducted a randomized controlled trial investigating the use of deciduous autologous tooth stem cells (hDPSCs) for regenerating dental pulp in injured teeth. This study involved 40 patients with traumatic pulp necrosis, where hDPSCs were implanted into the root canals, followed by sealing with mineral trioxide aggregate (MTA). The findings highlighted significant improvements in vascular and neural formation, as well as increased root length and width, suggesting that hDPSCs could be a viable option for salvaging young teeth and enhancing root development. This innovative approach is part of a broader trend in dental research focusing on tissue regeneration and the creation of a conducive microenvironment for healing [128].

### Clinical decision support tools

Clinical decision support tools (CDST) have also shown promise in improving the management of dental trauma. A. J. H. Huh (2022) evaluated the impact of CDSTs on the management of primary dentition traumatic injuries among pediatricians and dental professionals. The study demonstrated that both paper-based and mobile app CDSTs significantly improved diagnostic and management skills, particularly among medical students. The findings suggest that integrating CDSTs into educational programs could enhance the quality of dental trauma management and outcomes [90].

### Impact on quality of life

Basha et al. focused on special needs children, finding higher rates of trauma among those with cerebral palsy and obesity. The study by Aldrigui et al. linked dental trauma and caries to a negative impact on children’s oral health-related quality of life, underscoring the importance of early preventive measures and parental education. Aldrigui et al. assessed the impact of dental caries, traumatic dental injuries, and malocclusion on the oral health-related quality of life (OHRQoL) of preschool children [129]. Results showed that cavitated lesions and TDIs negatively affected OHRQoL, while malocclusion did not. Cavitated posterior teeth were particularly impactful. Socio-demographic factors such as lower income and mother’s education level were linked to caries prevalence. Parental perception of poor oral health correlated with negative OHRQoL. The study emphasizes the need for addressing oral health issues early to mitigate their impact on children and families [130].

### Limitations and future directions

Despite the comprehensive nature of this review, several limitations must be acknowledged. The inclusion criteria limited the studies to clinical trials and case series, potentially excluding valuable data from other study designs. Additionally, the reliance on published literature may introduce publication bias, where studies with positive outcomes are more likely to be published than those with negative or inconclusive results. Furthermore, the quality assessment using the ROBINS tool, although rigorous, is subject to reviewer interpretation and may introduce subjective bias [129].

These limitations could affect the interpretation of the results. For instance, the variability in study designs, sample sizes, and follow-up periods among the included studies may influence the consistency and generalizability of the findings. Moreover, the potential for publication bias suggests that the actual effectiveness of some interventions may be overestimated.

To address these gaps, future research should focus on large-scale, multicenter randomized controlled trials with standardized protocols to ensure consistency and comparability of results. Additionally, studies exploring the long-term outcomes of various treatment modalities and their impact on the quality of life in children are warranted. Incorporating a broader range of study designs, including observational studies and qualitative research, could provide a more comprehensive understanding of dental trauma management. Furthermore, efforts should be made to minimize publication bias by encouraging the publication of all research outcomes, regardless of the results [131].

In conclusion, while this review provides valuable insights into the management of dental trauma in children, addressing the identified limitations and biases through future research will enhance the robustness and applicability of the findings, ultimately improving clinical practice and patient outcomes [132–134].

The following images show some of the most common cases of dental trauma, especially in children and the front teeth. Early intervention can help manage pain and prevent further complications, especially when it comes to deciduous teeth as they are linked to the development of permanent teeth [135–137]. Depending on the severity, treatment might include repositioning of the teeth, monitoring for pulp vitality, or even extraction if the teeth are not salvageable. In less serious cases, reconstruct the fractured portion of the tooth as soon as possible in order to protect the tooth from excessive sensitivity and pain [138].

#### Case series

##### Case 1

**Fig. 4:**
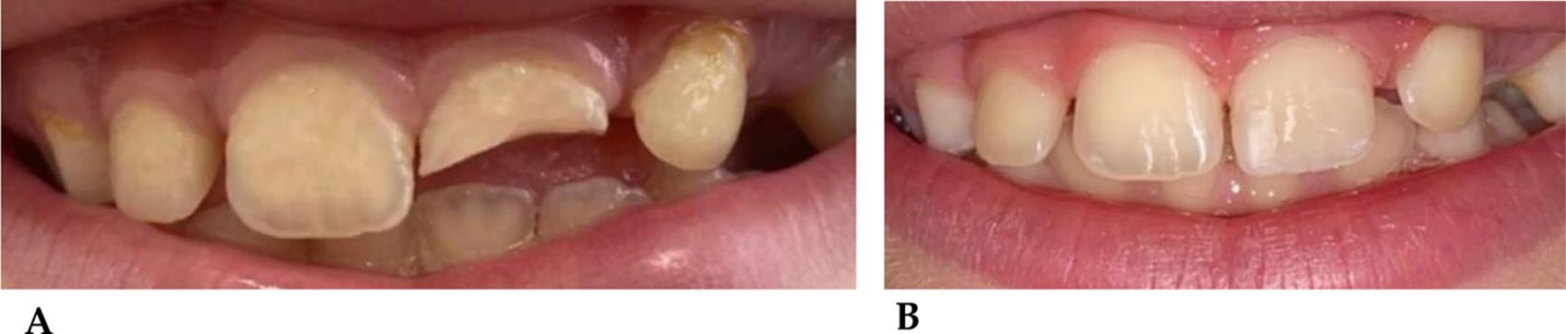
**A**: Crown fracture of tooth element 2.1, before reconstruction. **B**: Tooth after reconstruction in aesthetic material after 2 h from the accident

(Fig. 4A-B). Class II fracture, according to Ellis’ classification, of tooth element 2.1 in 8-year-old mixed dentition patient following accidental fall with bicycle [139].

##### Case 2

**Fig. 5:**
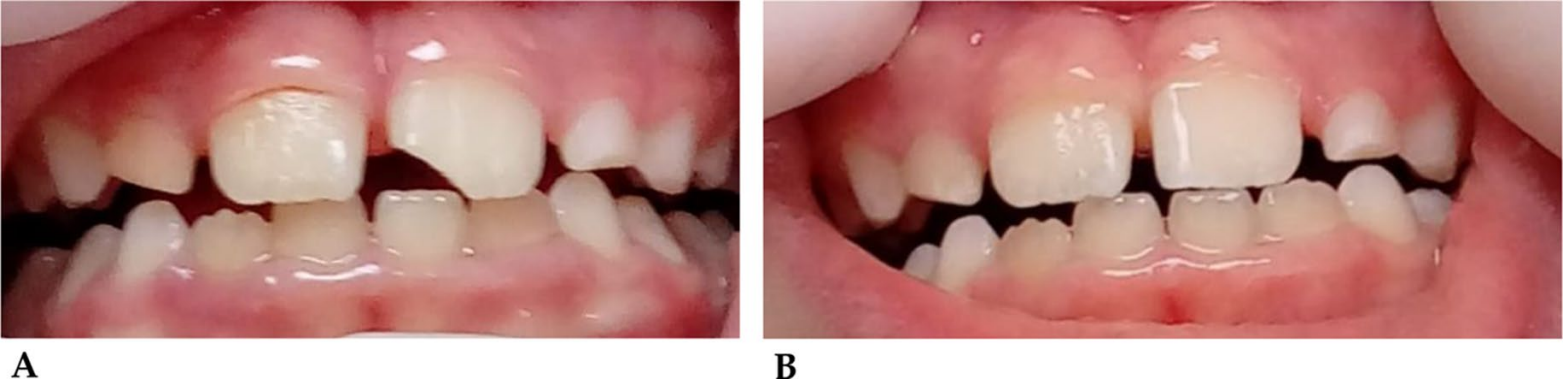
**A**: Dental element 2.1 fractured. **B**: Element 2.1 after reconstruction in aesthetic material made the following day

(Fig. 5A-B). Class II Ellis fracture of tooth element 2.1 in a 7-year-old girl in mixed dentition following a fall from the couch while playing with her little brother [140].

##### Case 3

**Fig. 6:**
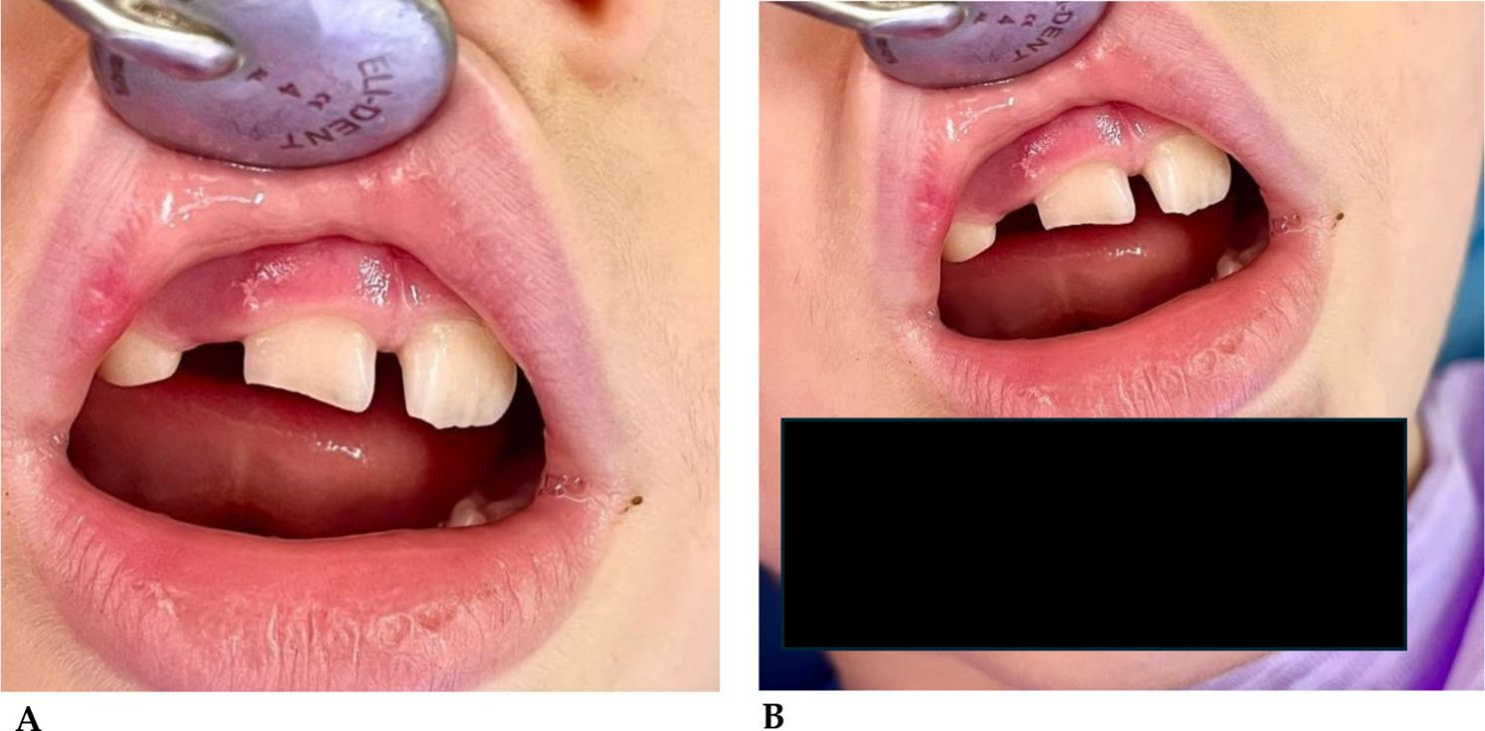
**A**: Marginal fracture of dental element 1.1. **B**: Dental element reconstructed with aesthetic material

(Fig. 6A-B). Element 1.1 with marginal first-class Ellis fracture in a 7-year-old boy in mixed dentition playing with a classmate [141].

##### Case 4

**Fig. 7:**
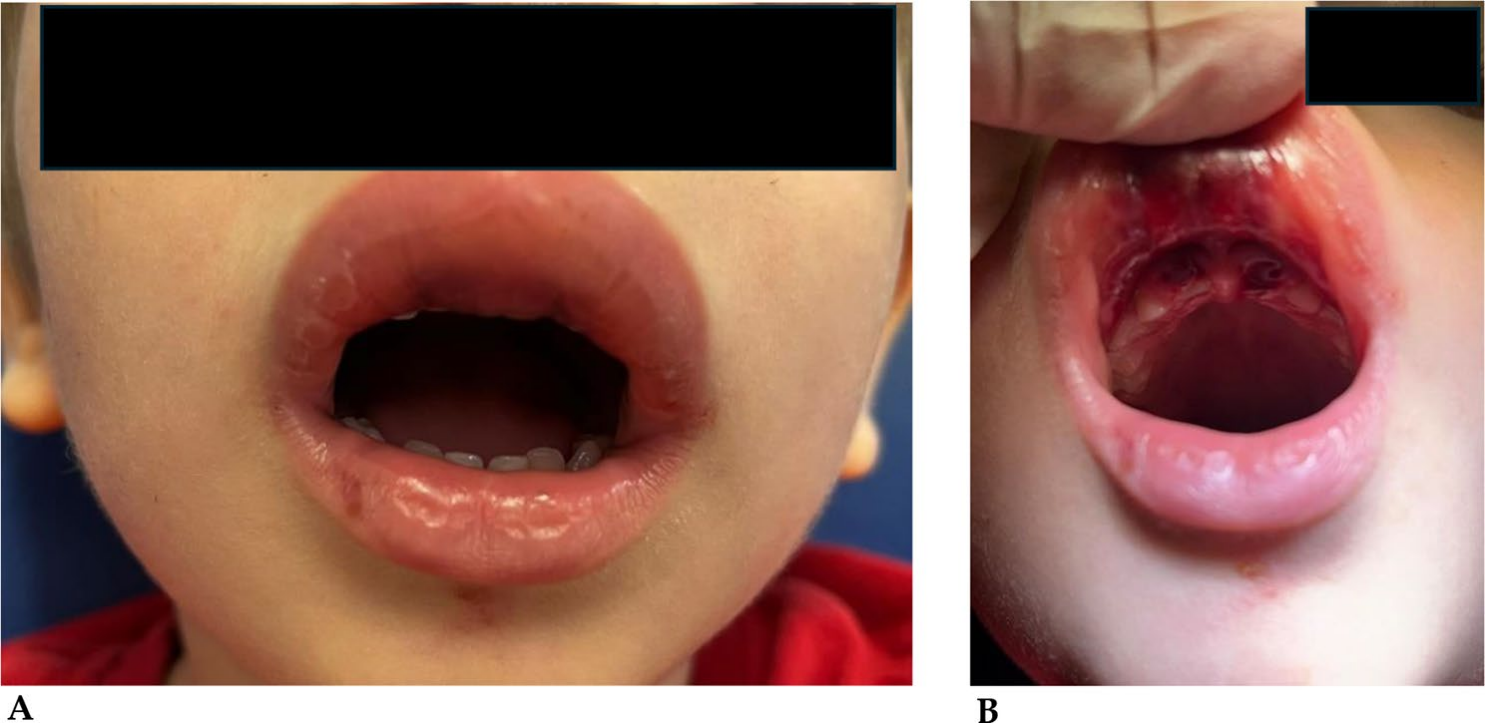
**A**: Swelling of the periooral tissues. **B**: Evident upper deciduous central incisor intrusion

(Fig. 7A-B). A 5-year-old boy suffered a bicycle accident, which resulted in the intrusion of the upper central deciduous incisors and swelling of the perioral tissues [33].

##### Case 5

**Fig. 8:**
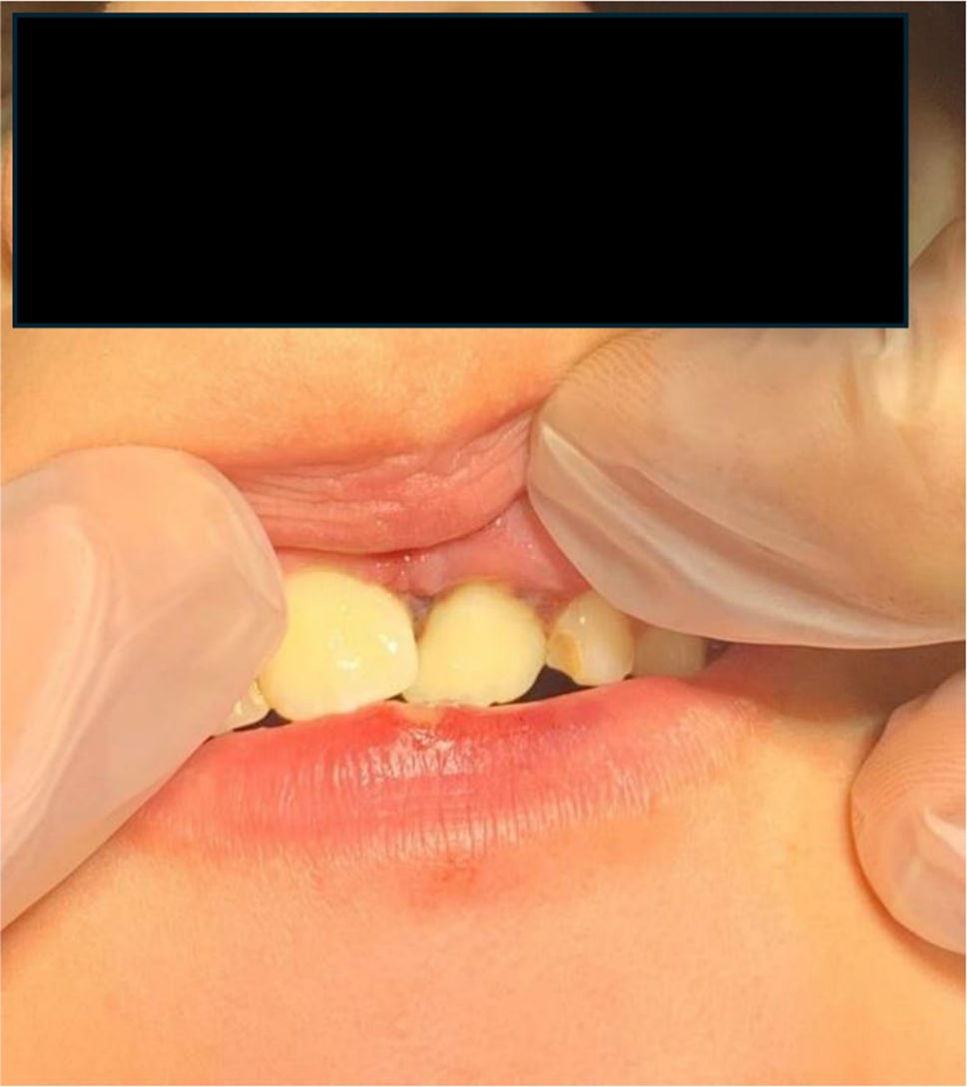
Evident palatization of the incisor immediately after the accident

(Fig. 8). Palatal dislocation of element 2.1 in a 4-year-old boy playing at home [142].

##### Case 6

**Fig. 9:**
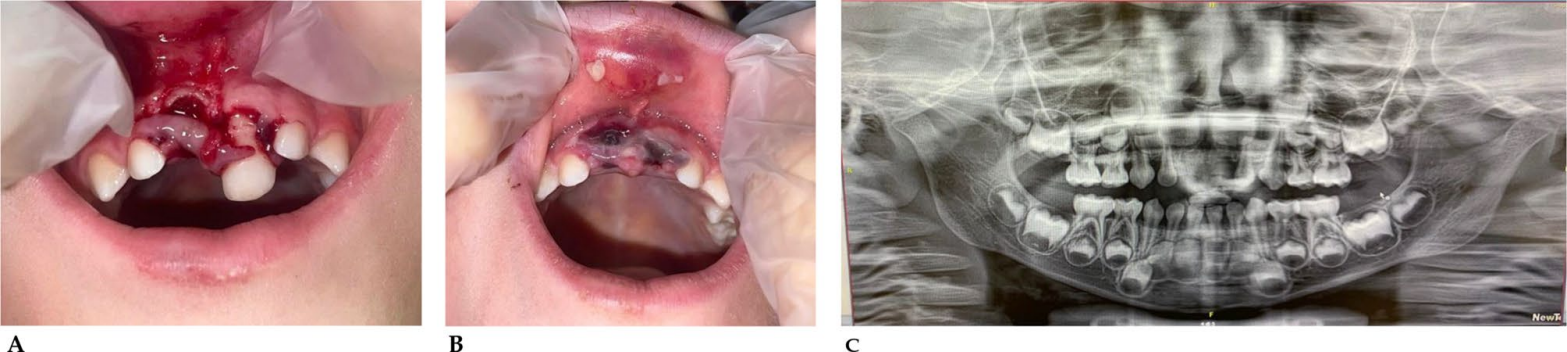
**A**: Loss of tooth 5.1 and dislocation of tooth 6.1. **B**: Fragments of tooth 5.1 in the upper lip. **C**: Panoramic x-ray of the patient’s dental arches

(Fig. 9A-B). The following images show the oral trauma of a 4-year-old girl who is slammed on the table. The trauma resulted in the loss of tooth 5.1 and dislocation with major extrusion of tooth 6.1. Fragments of tooth 5.1 were found in the vestibular area of the upper lip [143, 144].

##### Case 7

**Fig. 10:**
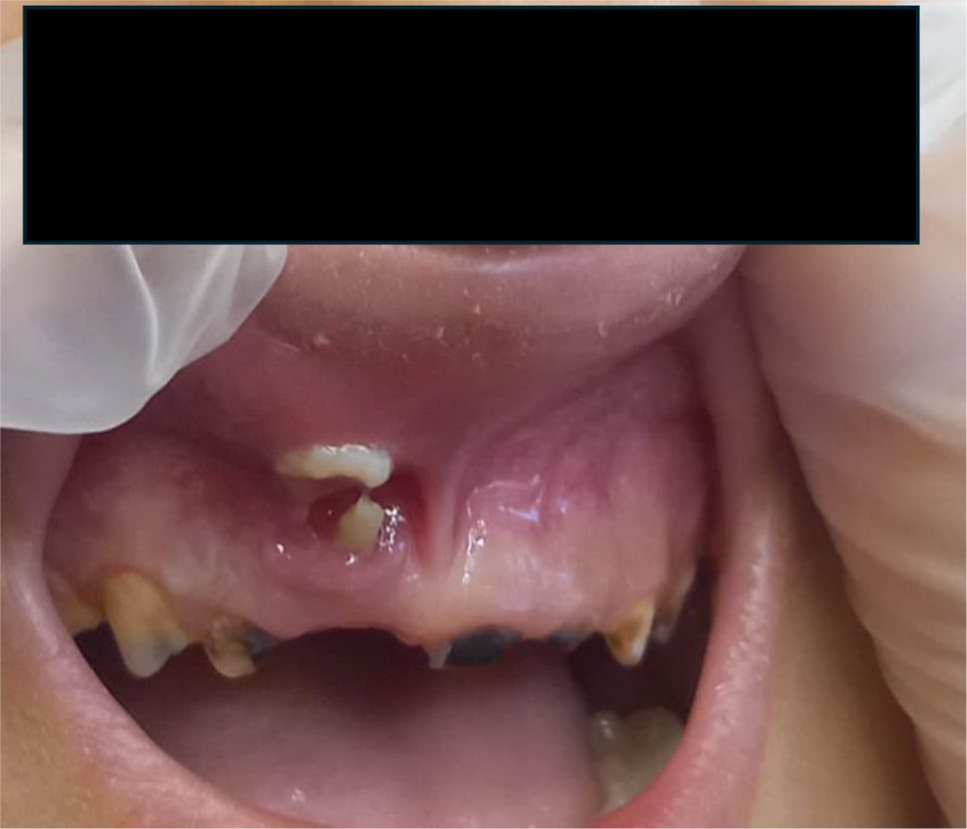
Intrusion and fracture of the deciduous right upper central incisor and gingival area swelling

(Fig. 10). Child who suffered significant dental trauma while playing with friends in a public playground. Visible signs are intrusion of the deciduous right upper central incisor with fracture. The impact pushed part of the tooth into the gum tissue. There is also swelling around the gingival area, indicating soft tissue trauma, consistent with a recent accident. There is presence of redness and bleeding in the gingival area. The trauma could also impair the development or eruption of the underlying permanent teeth.

In conclusion, the reviewed literature collectively underscores the complexities and long-term consequences of dental trauma in children. Advances in stem cell therapy, novel pulpotomy materials, and clinical decision support tools represent significant strides in improving treatment outcomes. Regular follow-ups, prompt interventions, and educational programs for parents, teachers, and healthcare providers are crucial in mitigating the adverse effects of dental trauma and ensuring better oral health for children. Continued research and longitudinal studies are essential to refine these approaches and validate their efficacy in diverse populations.

## Conclusions

Dental trauma in deciduous teeth is a prevalent and significant issue in pediatric dentistry, necessitating prompt and appropriate management to mitigate long-term consequences. This review consolidates current knowledge on the prevalence, types, management, complications, and prevention of dental trauma in primary teeth, aiming to inform clinical practice and future research. Common trauma causes include falls, sports injuries, and accidents involving objects or bicycles. Early diagnosis and intervention are essential to preserve the vitality of injured teeth and prevent complications. Immediate management of dental trauma is critical to improve outcomes. Reattaching fractured tooth fragments, performing root canal therapy, and reimplanting avulsed teeth within two hours are vital steps in the acute phase. It is important to have conservative approaches and to monitor, with follow-up care, for late complications. Long-term management often involves innovative treatments such as stem cell therapy and novel pulpotomy materials. These findings underscore the importance of integrating early intervention strategies and conservative approaches into clinical practice. Policymakers should emphasize preventive measures and education programs to reduce the incidence of dental trauma in children. Adopting these evidence-based recommendations can enhance the quality of care for pediatric dental trauma, ensuring better long-term oral health outcomes for young patients.

## Data Availability

Data is provided within the manuscript or supplementary information files.

## Declarations

### Disclaimer

The statements, opinions and data contained in all publications are solely those of the individual author(s) and contributor(s) and not of MDPI and/or the editor(s). MDPI and/or the editor(s) disclaim responsibility for any injury to people or property resulting from any ideas, methods, instructions or products referred to in the content.

## Abbreviations

CDST: Clinical decision support tool
CH: Calcium hydroxide
hDPSC: Autologous tooth stem cells from deciduous teeth
iRoot BP Plus: Innovative BioCeramic (Vancouver, BC)
MTA: Mineral trioxide aggregate
OHRQoL: Oral health-related quality of life
TDI: Traumatic dental injurie
